# Epidemiology of knife carrying among young British men

**DOI:** 10.1007/s00127-021-02031-x

**Published:** 2021-01-27

**Authors:** Jeremy Coid, Yingzhe Zhang, Yamin Zhang, Junmei Hu, Lindsay Thomson, Paul Bebbington, Kamaldeep Bhui

**Affiliations:** 1 Brain Research Center and Mental Health Center, West China Hospital of Sichuan University, 28th Dianxin South West Street, Wuhou District, Chengdu, 610041 People’s Republic of China; 2 Wolfson Institute of Preventive Medicine, Queen Mary University of London, London, UK; 3 West China School of Public Health, Sichuan University, Chengdu, Sichuan China; 4 West China School of Basic Medical Sciences and Forensic Medicine, China, Forensic Psychiatry, Sichuan University, Chengdu, China; 5 Forensic Psychiatry, University of Edinburgh, Edinburgh, UK; 6 University College London, London, UK

**Keywords:** Knife-carrying, Violence, Psychiatric morbidity, Ecological model, Socioeconomic deprivation

## Abstract

**Purpose:**

Knife carrying has caused considerable public concern in the UK. But little is known of the epidemiology and characteristics of men who carry knives. We investigated associations with socioeconomic deprivation, area-level factors, and psychiatric morbidity.

**Methods:**

Cross-sectional surveys of 5005 British men, 18–34 years, oversampling Black and Minority Ethnic (BME) men, lower social grades, and in London Borough of Hackney and Glasgow East. Participants completed questionnaires covering violent behaviour and psychiatric morbidity using standardised self-report instruments. Socioeconomic deprivation measured at small area level.

**Results:**

Prevalence of knife carrying was 5.5% (4.8–6.9) and similar among white and BME subgroups. However, prevalence was twice the national rate in Glasgow East, and four times higher among Black men in Hackney, both areas with high levels of background violence and gang activity. Knife carrying was associated with multiple social problems, attitudes encouraging violence, and psychiatric morbidity, including antisocial personality disorder (AOR 9.94 95% CI 7.28–13.56), drug dependence (AOR 2.96 95% CI 1.90–4.66), and paranoid ideation (AOR 6.05 95% CI 4.47–8.19). There was no evidence of a linear relationship with socioeconomic deprivation.

**Conclusion:**

Men who carry knives represent an important public health problem with high levels of health service use. It is not solely a criminal justice issue. Rates are increased in areas where street gangs are active. Contact with the criminal justice system provides opportunity for targeted violence prevention interventions involving engagement with integrated psychiatric, substance misuse, and criminal justice agencies.

## Introduction

Firearms account for the majority of intentional deaths in young men worldwide, mainly due to their accessibility. In the UK, however, knife crime accounts for more deaths than firearms, because firearm accessibility has been strictly and successfully controlled. Nevertheless, use and carrying of knives by young people is perceived as a growing problem in the UK. The British Medical Association has called for knife crime to be tackled as a public health concern [1] and politicians have proposed that healthcare professionals be legally responsible for identifying and reporting perpetrators of knife crime [2]. However, disproportionate media reporting of a “violence epidemic” may have over-estimated the increase in violence [3]. Furthermore, serious violence, including murder, declined internationally over the past 2 decades [4]. In UK, rates of violence-related injury remained lower during 2019 than the mid-2000s. Changes in police recording of offences may have also over-inflated these perceived changes. However, a small increase in deaths of men involving knives or sharp instruments occurred in England and Wales during 2018 [5], and persisted into 2019, prompting continuing media and public concern.

Little is known of the personal characteristics or epidemiology of men who carry knives in the UK. If these recent, modest increases in national rates are due to isolated pockets of violence in one region or city, this would be of concern. It would suggest a more targeted public health approach is needed in certain localities rather than nationwide campaigns or criminal justice actions [3]. A controversial response to knife carrying has been police stop-and-search policy, with black people in the UK nine times more likely to be stopped and searched than white people [6]. In London, however, where stop and searches are highest, more victims and perpetrators of homicides involving knives are of black and minority ethnic (BME) background. It has been suggested this may be related to an upsurge of gang-related violence in these communities [7]. However, London contrasts with observations of a marked decline over the past decade of violence by teenage males, together with homicide rates and gang violence observed in Scotland, previously the highest in Europe, particularly in Glasgow [8].

More information is needed on men who carry knives to identify whether knife carrying is a significant public health problem that warrants public concern. Furthermore, whether there are associations between ethnicity, unemployment, and area level effects. There are no previous studies of knife carrying to indicate whether psychiatric morbidity is associated.

The aims of this paper were to investigate (1) prevalence of young British men who carry knives and associations of knife carrying with demographic factors, including ethnicity, socioeconomic status, area-level effects, and specific geographical locations known to be associated with high levels of violence and gang activity, (2) associations between carrying knives and attitudes towards violence, (3) associations with psychiatric morbidity.

## Method

### Data collection

This study has been previously described [9]. The survey was carried out in 2011 based on random location sampling. Individual sampling units (census areas of 150 households) were randomly selected within British regions in proportion to their population to derive a representative sample of young men (18–34 years) from England, Scotland, and Wales. There were four additional, boost surveys, including young BME men, and those from lower social grades and, London Borough of Hackney and Glasgow East, Scotland, output areas characterised by high levels of violence and gang activity. The same sampling principles applied to each survey type.

The self-administered questionnaire piloted in a previous survey was adapted and informed consent obtained from respondents. Respondents completed pencil and paper questionnaires in privacy and were paid £5 for participation.

### Survey measures

The Psychosis Screening Questionnaire [10] described five symptoms and screened participants for psychosis when ≥ 2 criteria were met. The Hospital Anxiety and Depression Scale [11] was used to define Anxiety and Depression based on scores of > 11 in the past week. Scores > 20 on the Alcohol Use Disorders Identification Test [12], and scores > 25 on the Drug Use Identification Test [13] were used to identify alcohol or drug dependence, respectively.

Questions from the Structured Clinical Interview for DSM-IV Personality Disorders Screening Questionnaire [14] identified Antisocial Personality disorder (ASPD) when 3 or more of 7 items for adult antisocial personality were present, and Conduct disorder when 3 or more of 15 items before age 15 years.

At household-level, we included quintiles of area-level scores of Index of Multiple Deprivation (IMD) which measures levels of deprivation in small areas called ‘lower layer super output areas’ [15].

### Violence, violent attitudes, and child maltreatment

All participants were questioned about violent behaviour using questions from previous UK surveys [16, 17].

*Characteristics of violence* They were asked ‘‘Have you been in a physical fight, assaulted, or deliberately hit anyone in the past 5 years’ and if they had carried a knife. They were asked about outcome of violence, victims, number of incidents, whether violence occurred at sporting events, involved gang fights, whether they were gang members, and if they had ever used a weapon in a fight,

*Reasons for violence* They were asked whether violence was instrumental (to obtain money, drugs or sex), they had deliberately looked for a fight, often ruminated about violence, found violence exciting, easily lost their temper, became violent if disrespected, would typically obtain a weapon and look for someone who had threatened them.

*Victimization* They were asked if they had been a victim of violence, feared violent assault, experienced domestic violence, sexual assault.

*Criminality* They were asked about previous criminal convictions for violence and robbery, whether imprisoned, could easily obtain a firearm, and whether friends encouraged them in committing crimes.

*Adverse childhood experiences* They were asked if they had witnessed violence in their home, physical, sexual abuse, or neglect, been in care, or experienced a serious injury before age 16.

*Life events and daily living* We asked about contemporary factors such as whether they had a close relationship, had moved home in the preceding year, had a separation or divorce; been fired from their job, had serious money problems, no educational qualifications, were not in education employment or training, had experienced life-threatening injury or homelessness since the age of 16.

### Statistical analysis

We compared demographic characteristics of men carrying knives compared with the rest. We describe the distributions knife-carrying across the booster and main surveys. We undertook logistic regression modelling to test for associations. We tested small area level effects of socioeconomic deprivation and social status (unemployed), separately in the main and combined booster surveys, using quintiles of the Index of Multiple Deprivation (IMD) scores [15]. We then tested associations with risk factors for violence, childhood and adulthood victimization and trauma, and live events, and psychiatric morbidity, which were adjusted for demographic influences.

The study was approved by Queen Mary University of London ethics committee. All analyses were performed using SPSS 25.0.

## Results

### Demography and sampling

The weighted sample included 5005 men, 18–34 years of age: 1915 (38.3%) main survey; 1017 (20.3%) BME sample; 596 (11.9%) lower social classes; 712 (14.2%) Hackney; and 765 (15.3%) Glasgow East. Of the total sample, 381 (7.6%) reported carrying a knife in the past 5 years. Table 1 shows men who carried knives were younger, UK-born, single, unemployed, and Black, but fewer of south Asian origin. More were in the boost surveys of lower social classes, Hackney, and Glasgow East, but not BME men. Knife carrying showed highest prevalence in Hackney and Glasgow East. In the main sample, the overall population rate for young men 18–34 years based on the main representative survey of England, Scotland and Wales was 5.5% (4.8–6.9), with the rate for white men 5.8% (4.5–7.2). The representative BME boost indicated there were no significant differences between White, Black (5.9%, 3.6–8.2), or South Asian (5.1%, 2.9–7.3) men at the population level.

**Table 1:** Demographic characteristics of men who have carried knife and others (*n* = 5005)

Characteristic	Other men *n* = 4624	Carried knife *n* = 381	OR	95% CI
	*n* (%)	*n* (%)		
Non-UK born	641 (14.2)	33 (8.7)	0.59**	0.41–0.85
Single	2833 (61.9)	268 (70.7)	1.48***	1.18–1.86
Unemployed	1679 (37.1)	206 (57.2)	2.27***	1.83–2.82
*Ethnicity*				
White (reference)	2949 (63.9)	243 (63.8)	Ref	
Black	635 (13.8)	80 (21.0)	1.51**	1.15–1.97
South asian	964 (20.9)	51 (13.4)	0.63**	0.46–0.86
Other	69 (1.5)	7 (1.8)	1.15	0.51–2.60
*Survey type*				
Main (reference)	1,810 (39.1)	105 (27.7)	Ref	
Ethnic minorities	959 (20.7)	58 (15.2)	1.04	0.75–1.44
Lower social classes	544 (11.8)	52 (13.6)	1.63**	1.15–2.30
London, Hackney	630 (13.6)	82 (21.5)	2.22***	1.64–3.00
Glasgow East	681 (14.7)	84 (22.0)	2.10***	1.56–2.84

	Mean (SD)	Mean (SD)		
Age (years)	26.27 ± 4.97	25.27 ± 5.28	0.96***	0.94–0.98

*p < 0.05

**p < 0.01

***p < 0.001

Table 2 shows effects of unemployment and socioeconomic deprivation measured at small area level for (1) main survey (2) total combined sample. Main survey participants were stratified into five levels with equal numbers according to IMD scores where they lived. The same quintile ranges previously created were then applied to the combined sample, including boost surveys. Findings for the main survey indicated that, adjusted for age, quintiles 3–5 showed similar prevalence. All were significantly higher than reference category of men from least deprived areas, but not quintile 2. Following adjustment for unemployment, all three quintiles with higher IMD scores still showed significantly higher odds of association for knife carrying compared to reference quintile 1. There was no gradient in odds of association observed between quintiles 2–5 before or after adjustment in either the main or all surveys combined.

**Table 2:** Effects of small area level socioeconomic deprivation and social status (unemployment) on knife carrying

Socioeconomic deprivation (main survey) *n* = 1916	Other men *n* = 1810	Carried knife *n* = 106 Model 1			Carried knife *n* = 106 Model 2	
	*n* (%)	*n* (%)	OR	95% CI	AOR	95% CI
1 Least deprived, *n* = 384	377 (20.8)	7 (6.6)	Ref	–	Ref	–
2, *n* = 385	368 (20.3)	17 (16.0)	2.44	0.99–6.06	1.81	0.72–4.58
3, *n* = 384	351 (19.4)	33 (31.1)	5.24***	2.25–12.18	4.23***	1.80–9.95
4, *n* = 383	361 (19.9)	22 (20.8)	3.42**	1.42–8.22	2.91*	1.20–7.03
5 Most deprived, *n* = 380	353 (19.5)	27 (25.5)	4.27***	1.81–10.06	3.29**	1.37–7.85

Socioeconomic deprivation (all surveys) *n* = 5005	Other men *n* = 4624	Carried knife *n* = 381 Model 1			Carried knife *n* = 381 Model 2	
	*n* (%)	*n* (%)	OR	95% CI	AOR	95% CI
1 Least deprived, *n* = 459	446 (9.6)	14 (3.7)	Ref	–	Ref	–
2, *n* = 596	565 (12.2)	30 (7.9)	1.67	0.87–3.19	1.44	0.72–2.86
3, *n* = 758	708 (15.3)	50 (13.1)	2.25**	1.22–4.14	2.09*	1.10–3.96
4, *n* = 1249	1134 (24.5)	116 (30.4)	3.31***	1.87–5.86	3.32***	1.83–6.03
5 most deprived, *n* = 1943	1772 (38.3)	172 (45.0)	3.17***	1.81–5.55	2.87***	1.59–5.16

Model 1 is adjusted for age

Model 2 is adjusted for age and socioeconomic status (unemployment)

Table 3 shows independent associations between knife carrying and risk factors for future violence, including previous violence, criminality, and attitudes towards and characteristics of violence. All characteristics of previous violence showed strong positive associations with knife carrying.

**Table 3:** Independent Associations between knife carrying and individual and interpersonal factors (*n* = 5005)

	Other men *n* = 4,624	Carried knife *n* = 381	AOR	95% CI
	*n* (%)	*n* (%)		
Characteristics of violence				
Any violence in past 5 year	1264 (27.6)	344 (90.5)	23.82***	16.63–34.12
≥ 3 violent incidents	785 (17.4)	220 (65.1)	8.47***	6.56–10.93
Gang fight	99 (2.2)	163 (43.5)	30.63***	22.57–41.57
Intimate partner violence	111 (2.4)	83 (22.3)	11.79***	8.46–16.45
Instrumental violence	106 (2.3)	184 (48.8)	38.54***	28.65–51.85
Violence at sports events	199 (4.3)	155 (41.3)	14.01***	10.73–18.30
Used weapon in a fight	227 (5.0)	180 (49.6)	18.88***	14.46–24.66
Used weapon if threatened	277 (6.0)	198 (51.8)	24.19***	18.24–32.09
Outcome of violence				
Perpetrator injured	429 (9.4)	153 (41.1)	6.38***	4.99–8.15
Other person injured	523 (11.4)	173 (46.6)	6.68***	5.24–8.52
Police involved	280 (6.1)	115 (31.0)	6.80***	5.19–8.92
Victimization				
Fear violent victimization	752 (17.2)	145 (40.2)	3.07***	2.42–3.90
Assaulted and injured	641 (13.9)	125 (32.8)	2.79***	2.17–3.58
Attitudes to violence				
Excited by violence	191 (4.2)	187 (51.2)	22.76***	17.45–29.70
Violent ruminations	284 (6.5)	185 (52.6)	16.20***	12.54–20.93
Violent if disrespect	742 (18.2)	262 (74.2)	12.90***	9.85–16.90
Easily lose temper	470 (10.9)	213 (61.7)	13.05***	10.13–16.80
Looked for fight	139 (3.1)	173 (46.4)	24.48***	18.52–32.36
Associated criminality				
Previous violence conviction	237 (5.1)	115 (30.2)	7.50***	5.68–9.92
Previous robbery conviction	49 (1.1)	38 (10.0)	9.56***	6.03–15.15
Ever in prison	136 (2.9)	99 (26.1)	11.58***	8.42–15.91
Gang member	43 (1.0)	69 (19.2)	23.07***	14.89–35.75
Could obtain firearm	323 (7.4)	205 (62.5)	22.72***	17.30–29.83
Childhood victimization/trauma				
Bullying	1181 (25.5)	146 (38.3)	1.76***	1.40–2.22
Witnessed violence in home	408 (8.8)	160 (42.0)	7.53***	5.88–9.63
Sexual abuse	105 (2.3)	26 (6.8)	3.68***	2.33–5.82
Physical abuse	255 (5.5)	74 (19.4)	4.30***	3.18–5.80
Neglect	188 (4.1)	75 (19.7)	5.79***	4.26–7.87
Adult victimization/life events				
Domestic violence	110 (2.4)	36 (9.5)	4.59***	3.03–6.96
Sexual assault	43 (0.9)	18 (4.7)	5.02***	2.78–9.04
Life threatening injury	103 (2.2)	44 (11.5)	5.88***	3.95–8.75
Separation/divorce	340 (7.4)	49 (12.9)	1.96***	1.39–2.77
Fired from job	734 (15.9)	91 (23.9)	1.69***	1.29–2.21
Homelessness	258 (5.6)	116 (30.5)	7.26***	5.50–9.57
Serious money problems	683 (14.8)	184 (48.3)	5.92***	4.66–7.51
Daily living				
Close relationship	2490 (58.0)	139 (29.5)	0.47***	0.35–0.62
Moved home past year	1138 (25.1)	122 (33.1)	1.70***	1.33–2.16
No educational qualifications	529 (11.4)	105 (27.6)	2.46***	1.89–3.21
Not in education employment training	856 (19.2)	148 (40.9)	2.24***	1.62–3.10
Encouraged by friends into crime	294 (6.7)	155 (46.7)	10.62***	8.16–13.82

Adjusted for non-UK birth, being single, unemployment, ethnicity, age, and survey type

*p < 0.05

**p < 0.01

***p < 0.001

Table 3 also shows that men who reported carrying knives had experienced multiple childhood victimization, maltreatment and trauma, and adverse life events in adulthood. They were unlikely to be in close relationships. They were more likely to have moved house in the past year, no qualifications, were not in employment, education or training, and part of a criminal peer group.

Table 4 shows independent associations between psychiatric morbidity and carrying a knife. Following adjustments, there was no association with depression, but all other forms of psychopathology were significantly higher, particularly ASPD and conduct disorder. Men carrying knives were also significantly more likely than other men to report all forms of psychiatric service use. We investigated whether there were age trends in associations with psychiatric morbidity observed in Table 4. We found that two conditions showed a significant trend for increasing prevalence with age. First, anxiety disorder: 18–21 years, (*n* = 34, 28.6%); 22–25 years, (*n* = 23, 30.3%); 26–29 years, (*n* = 26, 39.4%); 30–34 years, (*n* = 60, 54.5%); (Chi-square trend = 19.11, *p* < 0.001). Second, drug abuse: 18–21 years, (*n* = 20, 17.7%); 22–25 years, (*n* = 15, 19.2%); 26–29 years, (*n* = 22, 32.4%); 30–34 years, (*n* = 34, 32.7%), (Chi-square trend = 9.82, *p* < 0.05). We investigated whether this corresponded to prevalence of gang membership in each age band, but there was no significant age trend for the latter (Chi-square trend = 1.26, NS).

**Table 4:** Independent Associations between knife carrying and psychiatric morbidity (*n* = 5005)

Measure	Other men *n* = 4624	Carried knife *n* = 381	AOR	95% CI
	*n* (%)	*n* (%)		
Psychiatric morbidity				
Anxiety^a^	597 (13.1)	143 (38.4)	1.10	0.75–1.61
Depression^a^	426 (9.4)	71 (19.3)	1.47	0.96–2.25
Alcohol dependence^a^	370 (8.2)	138 (39.3)	2.35***	1.65–3.33
Drug dependence^a^	80 (1.8)	91 (25.1)	2.96***	1.90–4.66
Antisocial personality disorder^a^	434 (9.8)	220 (62.1)	9.94***	7.28–13.56
Suicide attempt^b^	226 (5.0)	78 (21.5)	4.58***	3.38–6.21
Conduct disorder^b^	879 (19.4)	260 (69.3)	10.53***	8.18–13.55
Psychosis (PSQ ≥ 2)^a^	221 (4.9)	96 (26.2)	1.80**	1.19–2.74
PSQ items				
Hypomania ^c^	161 (3.5)	47 (12.3)	1.87**	1.20–2.92
Thought insertion^c^	112 (2.4)	40 (10.5)	1.22	0.73–2.05
Paranoia delusion^c^	301 (6.5)	143 (37.5)	6.05***	4.47–8.19
Delusional mood/perception^c^	261 (5.6)	83 (21.8)	1.26	0.84–1.90
Hallucinations	142 (3.1)	51 (13.4)	1.26	0.79–2.02
Psychiatric service use^b^				
Consulted medical practitioner	374 (8.1)	76 (20.2)	3.10***	2.32–4.13
Consulted psychiatrist or psychologist	82 (1.8)	36 (9.5)	5.00****	3.23–7.75
Psychiatric admission	145 (3.2)	45 (12.2)	3.50***	2.40–5.10
Psychotropic medication	174 (3.9)	51 (13.9)	3.31***	2.30–4.77

^a^Adjusted for other psychiatric morbidity outcomes, non-UK birth, being single, unemployment, ethnicity, age, and survey type

^b^Adjusted for non-UK birth, being single, unemployment, ethnicity, age, and survey type

^c^Adjusted for other psychosis outcomes, non-UK birth, being single, unemployment, ethnicity, age, and survey type

*p < 0.05

**p < 0.01

***p < 0.001

## Discussion

Given strong associations with physical harm and psychiatric morbidity, carrying knives should be a cause of concern for public health services. We found that 1 in 18 men age 18–34 years in Britain reported having carried a knife with personal characteristics indicating risks for future violence. We did not find higher rates of knife carrying among any specific ethnic group using representative samples of the general population. However, there may be specific area-level effects in Hackney for BME men and Glasgow east (where all participants were white) which require further investigation in similar, atypical locations with high rates. Men who carry knives were of lower social class. However, when we specifically investigated associations with socioeconomic deprivation (SED) at national level, there was no simple linear association. Knife carrying was less common in the 40% of areas characterised by lowest levels of SED, but showed similar rates across the remaining 60% and did not correspond to increasing levels of SED. They were also somewhat younger in the sample and there was a trend of declining prevalence with age. However, a sub-group showed persistence in the 30–34 years age group.

### Ecological model

**Fig. 1:**
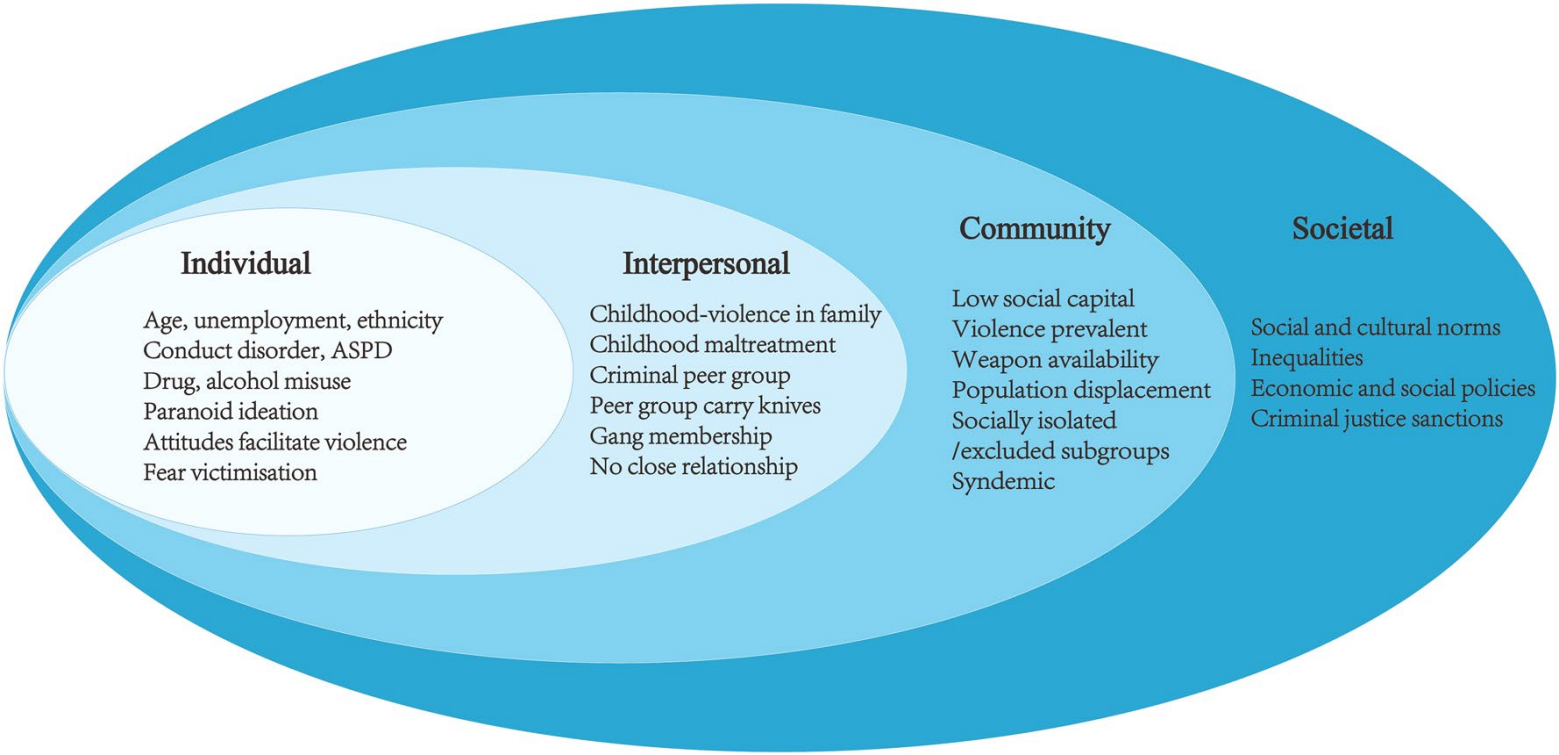
Ecological model of knife carrying

Men who self-reported that they had carried knives showed multiple problems of violence, criminality, adverse childhood experiences, educational and occupational disadvantage, traumatic life events, substance misuse, psychiatric morbidity and service use. These associated factors can be considered within an ecological model of violence [18] (see Fig. 1) in which a complex series of adverse, inter-related factors have impacted on these men over the life-course. Key individual biological and personal history factors include persistence of conduct disorder into adulthood as ASPD, the most prevalent psychiatric condition. Our study must therefore be compared to studies of younger persons where prevalence is much higher and the peak age of carrying knives is 14 years, but where the majority desist leaving a “hard core” with multiple interpersonal and individual level problems [19]. The latter correspond to our sample, with multiple antisocial behaviours and associated psychopathology including anxiety, suicidal behaviour and substance misuse [20–22]. Anxiety and drug misuse may have contributed to these men persisting in carrying knives into later age than would have been expected. Violence tends to show a progressive decline in prevalence at the population level from mid-teenage years onwards. However, our findings for carrying knives did not follow this trend, suggesting a marker for a more severe and persistent form of antisocial behaviour. The association with psychosis corresponds to previous findings for both violence [16] and gang membership [9] and was largely explained by symptoms of paranoid ideation. Meta-analysis has shown paranoid ideation is most strongly associated with violence at the population level [23].

The interpersonal experience of witnessing violence in the home in the ecological model (Fig. 1) was the most commonly reported childhood experience [24]. Childhood physical abuse is the most consistent predictor of violence, particularly when compounded by additional forms of maltreatment such as sexual abuse and neglect [25]. Bullying by peers corresponds to weapon carrying, including firearms [26]. Nevertheless, other individual factors should be considered in both childhood and adulthood, including intention to intimidate others, facilitate robbery, deliberately injure, or simply for perceived power and status that carrying a weapon provides [27].

Inability to maintain close relationships meant these men did not experience protective factors of a supportive intimate partner. Many were part of a criminal peer group. Although most men who carried knives were not gang members, this factor showed one of the strongest associations with knife carrying, corresponding to dramatic increase in violent and criminal behaviour observed after joining a gang [28].

Associations between socioeconomic deprivation and knife carrying were complex and did not show a simple linear relationship, questioning whether societal norms for this behaviour were largely independent of these factors. The observed trends suggest that men who carry knives are considerably fewer in more affluent areas, with no simple relationship shown with poverty. However, low social capital is frequently associated with SED and may have influenced knife carrying in Glasgow where communities have not shown similar resilience to the effects of poverty and deprivation as other cities in the UK [29].

Our cross-sectional method meant we could not conclude whether there had been epidemic spread of carrying knives and whether this had become a societal and cultural norm. Knife carrying appeared evenly distributed through 60% of areas in Britain compared to the 40% showing lowest levels of SED. SED was not associated with carrying knives in a Scottish survey of younger people [19]. Nevertheless, two urban locations, surveyed because of high levels of violence and gang activity, did show exceptional levels. This is of major concern and supports the notion that a small number of urban areas with high concentrations of young men with multiple problems could be vulnerable to epidemic spread, leading to high levels of knife crime and requiring targeted interventions in these areas [3]. Our cross-sectional method also meant we could not demonstrate epidemics of knife crime related to gang activity. Nevertheless, US gang-related murders are confirmed as having an epidemic-like process of social contagion, similar to infectious disease [30], and which could be applied to future study of knife-related crime in Britain.

Societal and cultural norms which create an environment that accepts or condones knife carrying and violence were not specifically measured in this study. Societal factors have implications for population-level preventive interventions, are usually broad factors that reduce inhibitions against violence, and in previous studies have included poverty, economic, social and gender inequalities, poor social security, low social capital, social and cultural norms, masculinity linked to violence, weak legal and criminal justice sanctions, weapon availability, and population displacement [18]. Our study did not support a key role for all these factors, including poverty. However, we have shown syndemic effects between psychosis, substance dependence, high risk sexual behaviour and crime and violence, with knife carrying as a component of the latter, in Hackney, east London. This disproportionally affected young Black men [31]. However, despite black men in Hackney showing a prevalence of knife carrying four times the national average, black men across Britain showed a slightly lower prevalence than white men suggesting that they had overcome disadvantages, with no overall association between knife carrying and ethnicity.

### Limitations

Our study has several limitations. Because knife carrying was measured at any time over the past 5 years, some participants may have ceased carrying knives by the time of survey. The cross-sectional method prevented firm conclusions on direction of association. The possibility that knife carrying in association with violent behaviour leads to psychiatric morbidity must therefore be considered, together with bi-directionality of many associations we have described. Furthermore, we were unable to investigate cohort effects of age on knife carrying.

Self-report may have underestimated true prevalence of knife carrying, because socially undesirable behaviours tend to be less frequently reported. Our definition of gang membership did not correspond to an accepted definition and was deliberately broad to avoid eliminating cases. However, self-reported gang membership is generally accepted as a key component.

Random location sampling does not provide detailed information on number of young men who declined to participate. However, because the method is based on the National Census, participants were identified and included according to representative strata and actual frequency in the population. This method has considerable advantages for investigating health-related behaviours such as violence and criminality.

### Implications

Knife carrying represents an extensive, closely related series of Public Health problems. Population-level interventions may be appropriate for juveniles but are not strongly supported for men 18–34 years on the basis of these findings. It is important that the fall in convictions for violent crime 2017–2018 in Scotland occurred predominantly among those age 13–19 years, with little change among those 25 years and above [8]. Early prevention strategies in schools have been recommended based on observations that young persons are more likely to desist, although many young persons who carry knives will leave school at the earliest opportunity [19].

It is unclear whether decline in knife-related crime in Scotland was related to any specific interventions. The Violence Reduction Unit of Police Scotland was established in 2005 at a time of rising homicide rates and is considered the most likely explanation [32]. The unit adopted a Public Health approach [18] but did not involve Public Health Agencies and was based on a successful programme implemented by police and social services in the USA [33]. Its aims were to reduce violence by working with health, education and social work agencies to achieve societal and individual attitudinal change by focusing on enforcement and contain and manage individuals who carry weapons and are involved in violent behaviour. Emphasis on enforcement was balanced by a rehabilitative approach in which desistance was rewarded with support to find employment, education, and healthcare, including treatment for substance abuse.

High levels of psychiatric morbidity indicate need for additional involvement of mental health in an integrated approach with criminal justice agencies. Although few mental healthcare professionals are currently trained to contribute to public mental health prevention programmes, young men who carry knives are already accessing mental health services according to our findings. Our findings also suggest that interventions should be targeted in specific geographical locations, particularly inner-London, where continuing trends of more knife crime suggest that stop-and-search policy has had little impact. Support for police from local communities was essential in development of the Public Health model in Scotland. However, factors of ethnicity may make this more difficult in English cities. Findings that Black men are no more likely to carry knives nationally indicate the need to target men primarily on the basis of their criminal activities, with histories of violence and gang membership. Further research is needed into why certain inner urban areas generate clusters of multiple risks, including knife carrying, high levels of violence, and gang activity, and factors which increase or impede the transmission of these public health problems between communities.
